# Periostin: a potent chemotactic factor for recruiting tumor-associated macrophage

**DOI:** 10.1007/s13238-015-0141-9

**Published:** 2015-03-11

**Authors:** Tiantian Wu, Qi Luo, Gaoliang Ouyang

**Affiliations:** 1 State Key Laboratory of Cellular Stress Biology, Innovation Center for Cell Signaling Network, School of Life Sciences, Xiamen University, Xiamen, 361102 China; 2 Department of Surgical Oncology, First Affiliated Hospital of Xiamen University, Xiamen, 361003 China

**Figure 1:**
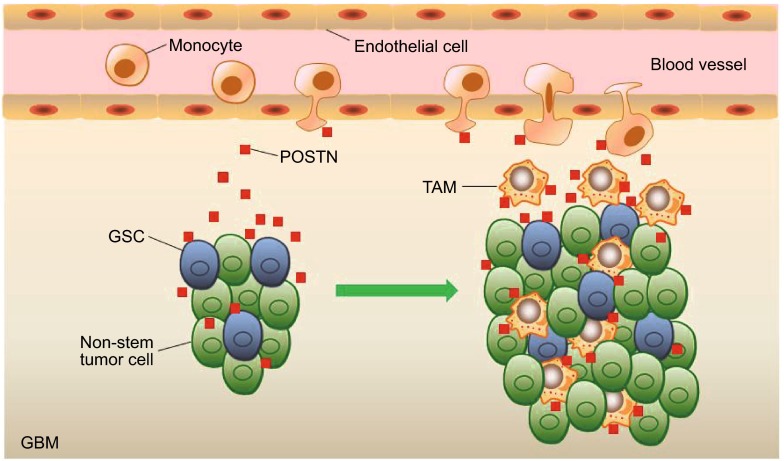
**Glioma stem cells (GSCs) secrete periostin (POSTN) to recruit and educate macrophages in glioblastoma (GBM)**. Tumor-associated macrophages (TAMs) are enriched in the POSTN-abundant perivascular niche in GBMs. GSC-secreted POSTN acts as a chemoattractant to recruit and maintain monocyte-derived M2 TAMs from the peripheral blood to promote GBM growth

Glioblastoma (GBM) is the most common malignant primary brain tumor with a low five-year survival rate. Evidence from experimental and clinical studies indicates that glioma stem cells (GSCs) contribute to GBM tumor growth, therapeutic resistance and relapse (Bao et al., 2006; Gilbertson and Rich, 2007). GSCs are enriched in a unique microenvironment known as the perivascular niche. Interestingly, a large number of tumor-associated macrophages (TAMs) are also distributed in the perivascular niche, indicating that crosstalk between GSCs and TAMs may have a critical role in GBM tumor progression. TAMs are abundant in most solid tumors and contribute to tumor progression in several ways, such as promoting invasion, angiogenesis and immunosuppression; however, the molecular link between TAM recruitment and GSCs has remained generally unclear. Writing in *Nature Cell Biology*, Zhou et al. provide new insights into where and how TAMs are recruited and educated by GSCs in GBMs (Zhou et al., 2015) (Fig. 1).

Using *in vitro* migration and invasion assays, Zhou et al. (2015) identified periostin (POSTN) as one of the most abundant GSC-secreted cytokines to potently attract macrophages. TCGA database analyses demonstrated that POSTN expression is negatively related to the survival of GBM patients. The authors further confirmed that POSTN is preferentially expressed and secreted by GSCs in human GBMs. Moreover, TAMs are enriched in POSTN-abundant regions in primary human GBM tissues, indicating that TAM density is positively related to POSTN level in human GBMs. Furthermore, knockdown of POSTN in GSCs significantly inhibits tumor growth and prolongs the survival of mice bearing GSC-derived GBM xenografts. Interestingly, silencing POSTN in GSCs also reduces TAM density by more than 65% in GSC-derived tumors. The authors further demonstrated that conditioned media from GSCs markedly attract more primed U937 macrophage-like cells than conditioned media from non-stem cancer cells do, whereas treatment with an anti-POSTN antibody or knockdown of POSTN in GSCs dramatically attenuates such a chemoattractant effect. PMA-primed U937 cells and TAMs in GBM xenografts and human primary GBM tumors express a POSTN receptor, integrin α_v_β_3_. POSTN can activate Akt in macrophages, and anti-α_v_β_3_ integrin antibody blocks this activation. Remarkably, treating primed U937 cells and GSC-derived GBM xenografts with integrin-inhibitory RGD peptide significantly attenuates POSTN-induced macrophage recruitment and decreases TAM density in GSC-derived GBM xenografts. Thus, POSTN recruits TAMs in GBM tumors via integrin α_v_β_3_ signaling.

TAMs in GBMs are currently thought to be either monocyte-derived macrophages from the peripheral blood or resident brain microglia. To determine the origin of TAMs in GBMs, Zhou et al. (2015) analyzed the specific macrophage markers in GSC-derived xenografts as well as in primary GBM tumors. The authors found that the majority of macrophages are resident microglia (CX3CR1^+^/CCR2^−^) in the normal mouse brain, whereas only CCR2^+^/CX3CR1^−^ macrophages, the monocyte-derived macrophages from the peripheral blood, are found in the GSC-derived GBM xenografts. Moreover, TAMs are CCR2^+^/CX3CR1^−^ in most human primary GBMs and are abundant around blood vessels both in GSC-derived xenografts and in primary GBM tumors, indicating that the recruited TAMs in GBMs are mainly monocyte-derived macrophages from the peripheral blood. The authors further used M1-specific markers (MHCII and CD11c) and M2-specific markers (Fizz-1, CD163 and Arg1) to determine which TAMs are recruited or maintained by GSC-derived POSTN. Knockdown of POSTN in GSCs markedly reduces M2 TAMs in GBM tumors, whereas overexpression of POSTN in GSCs promotes M2 TAM recruitment and augments xenograft growth. Furthermore, POSTN is also able to inhibit macrophage maturation. In addition, intracranial co-transplantation of M2 TAMs with GSCs significantly promotes tumor growth and shortens the survival of mice compared with the transplantation of GSCs alone. Mice co-transplanted with M2 TAMs and POSTN-silenced GSCs show a shortened survival relative to mice implanted with POSTN-silenced GSCs alone. Thus, M2 TAMs are recruited by GSC-derived POSTN and play a critical role in mediating POSTN-promoted GBM growth.

Although these findings provide new insights into how TAMs are recruited to promote GBM tumor growth, several questions remain unanswered. POSTN is a multifunctional matricellular protein that has an important role in regulating the inflammatory and tumor microenvironments (Conway et al., 2014; Liu et al., 2014; Wu and Ouyang, 2014). Apart from recruiting TAMs to promote GBM tumor growth, whether POSTN has a direct impact on remodeling the tumor microenvironment in GBMs, and especially the GSC perivascular niche, is unclear. As a Th2 cytokine, POSTN can be induced by TGF-β, IL-4, IL-13, IL-17, TNF-α, histamine, glucose and palmitate and can directly bind type 1 collagen, fibronectin, tenascin-C, BMP-1, Notch1 and itself, in addition to being regulated by Twist1, Twist2, STAT3, c-Jun, ChREBP and YY1 in the context of tissue injury, inflammation, fibrosis and/or tumors (Masuoka et al., 2012; Liu et al., 2014; Lu et al., 2014; Wu et al., 2014). Thus, the microenvironmental cues, transcription factors and microRNAs that directly induce or regulate POSTN expression in GSCs remain to be identified. GSCs have the ability to transdifferentiate into pericytes and endothelial cells in GBMs (Ricci-Vitiani et al., 2010; Wang et al., 2010; Cheng et al., 2013; Liu and Ouyang, 2013), but whether these GSC-derived pericytes and endothelial cells still secrete POSTN and have the potential to recruit TAMs, similar to GSCs, is unknown. It is also unclear whether GSCs can directly transdifferentiate into TAMs, at least in part. Interestingly, our recent work demonstrates that the activated hepatic stellate cells-derived POSTN might act as a chemoattractant factor to recruit macrophages into mouse acute and chronic fibrotic liver tissues (Huang et al., 2015). GSCs can secrete POSTN to recruit TAMs in GBMs, and POSTN knockdown significantly decreases TAMs in GSC-derived xenografts, but whether POSTN can act as a chemoattractant to recruit TAMs or other cells in other tumors remains to be determined. POSTN significantly promotes colon cancer cells metastasis to the liver by promoting the survival of cancer cells and endothelial cells (Bao et al., 2004). In breast cancer, fibroblast-derived POSTN is crucial for breast cancer stem cell expansion during metastatic colonization in lung (Malanchi et al., 2012; Wang and Ouyang, 2012), which is very different from the function of GSC-derived POSTN in GBMs. This difference and the effects of GSC-derived POSTN on non-stem cancer cells in tumor bulk still need to be investigated.

The finding of Zhou et al. (2015) that targeting POSTN significantly inhibits GBM tumor growth, mainly by reducing TAM recruitment and inducing massive apoptosis in GSC-derived xenografts, indicates that targeting POSTN-mediated TAM recruitment may improve GBM treatment. Interestingly, continuous anti-macrophage therapy with CCL2 neutralization by blocking the binding of CCL2 to CCR2-expressing monocytes impairs monocyte infiltration into primary mammary tumors and lung metastases in mice (Qian et al., 2011). However, a recent report demonstrated that cessation of anti-CCL2 therapy unexpectedly results in accelerated lung metastasis in mice (Bonapace et al., 2014). Considering that silencing POSTN expression has no marked direct impact on the GSC population, cancer cell proliferation or vascular density in tumor bulk, we do not know whether TAM recruitment will rapidly rebound in tumors and lead to lethal relapse after withdrawal of POSTN-targeting therapy. Therefore, targeting POSTN-mediated TAM recruitment may be more effective in fighting GBMs when used in synergy with other therapies.
